# Supplementary material for: Evaluating the predictive power of combined gene expression dynamics from single cells on antibiotic survival
Source: mSystems. 2025 May 20;10(6):e01588-24. doi: 10.1128/msystems.01588-24 (PMC12172483; doi:10.1128/msystems.01588-24)
Supplement: Supplemental Information — Supplemental figures, tables, movie captions, text, and references. [file msystems.01588-24-s0001.pdf]

Evaluating the predictive power of combined gene expression dynamics from single cells on antibiotic survival

Supplementary Information:

1. Supplementary Figures
2. Supplementary Tables
3. Supplementary Movie Captions
4. Supplementary Text
5. Supplementary References

Supplementary Figures

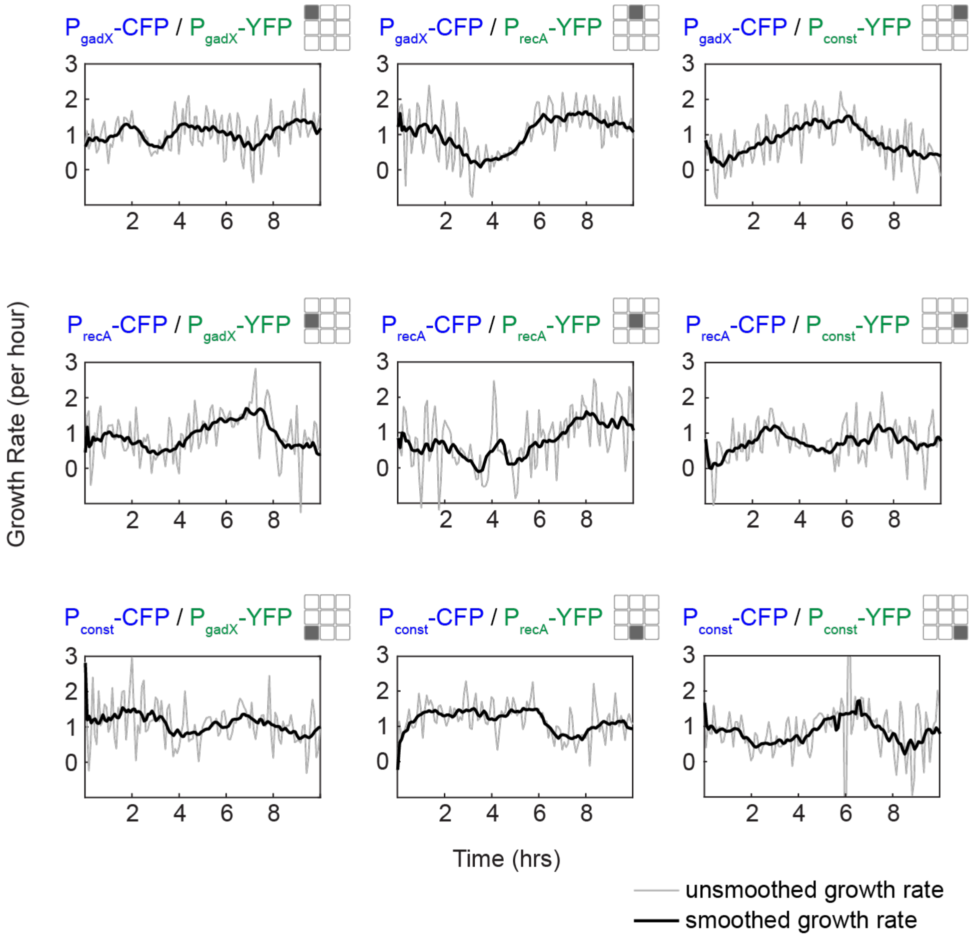

Figure S1. Smoothing growth rate. Growth rates of representative cells. Raw growth rate values extracted from the image analysis process are shown with a thin grey line, and smoothed growth rate with a 1 hour window are shown in a thick black line.

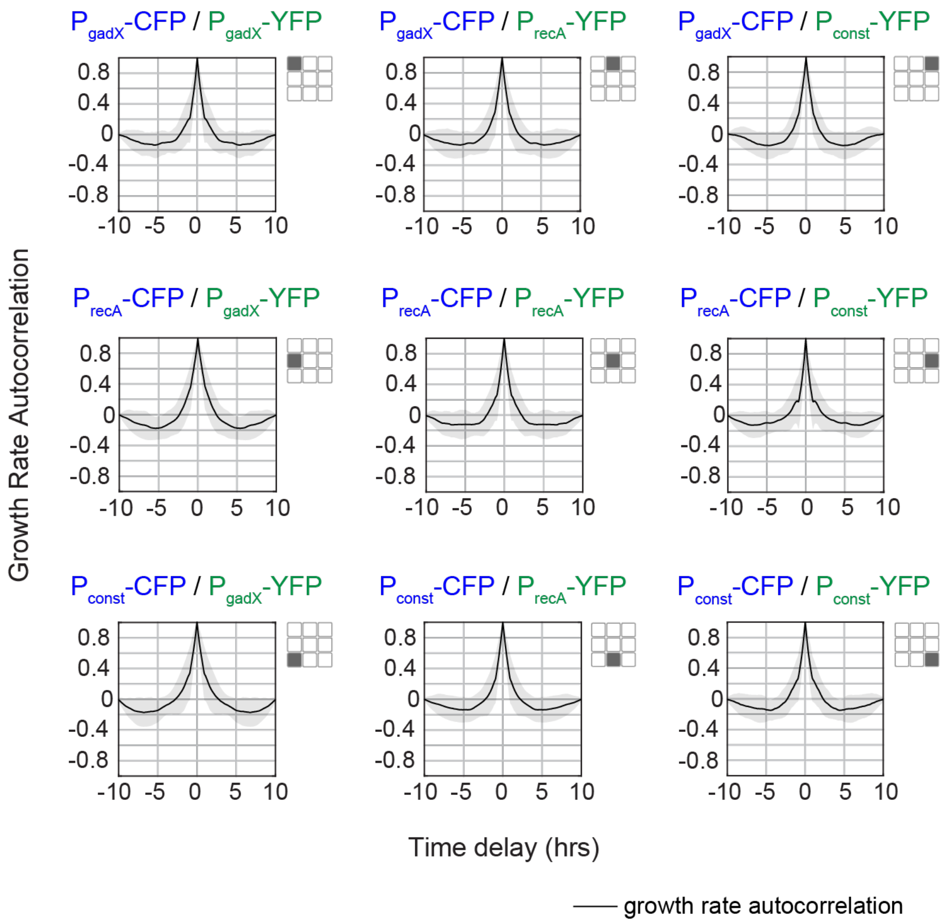

Figure S2. Growth rate autocorrelations. Autocorrelation of growth rates for each of the dual reporter strains. Line represents average of all cells and shaded region is plus or minus one standard deviation.

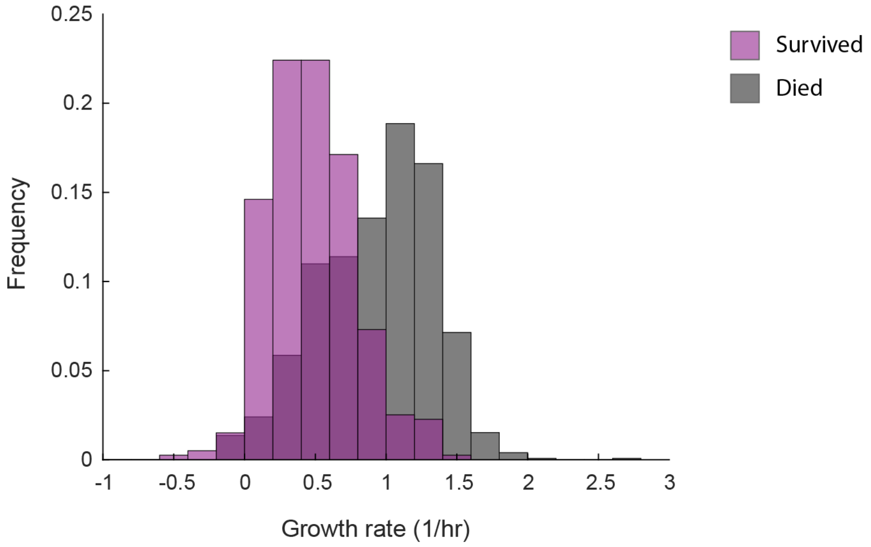

Figure S3. Growth rate and ciprofloxacin survival. Histogram showing the growth rate of cells that survived (purple) versus died (grey) after ciprofloxacin exposure.

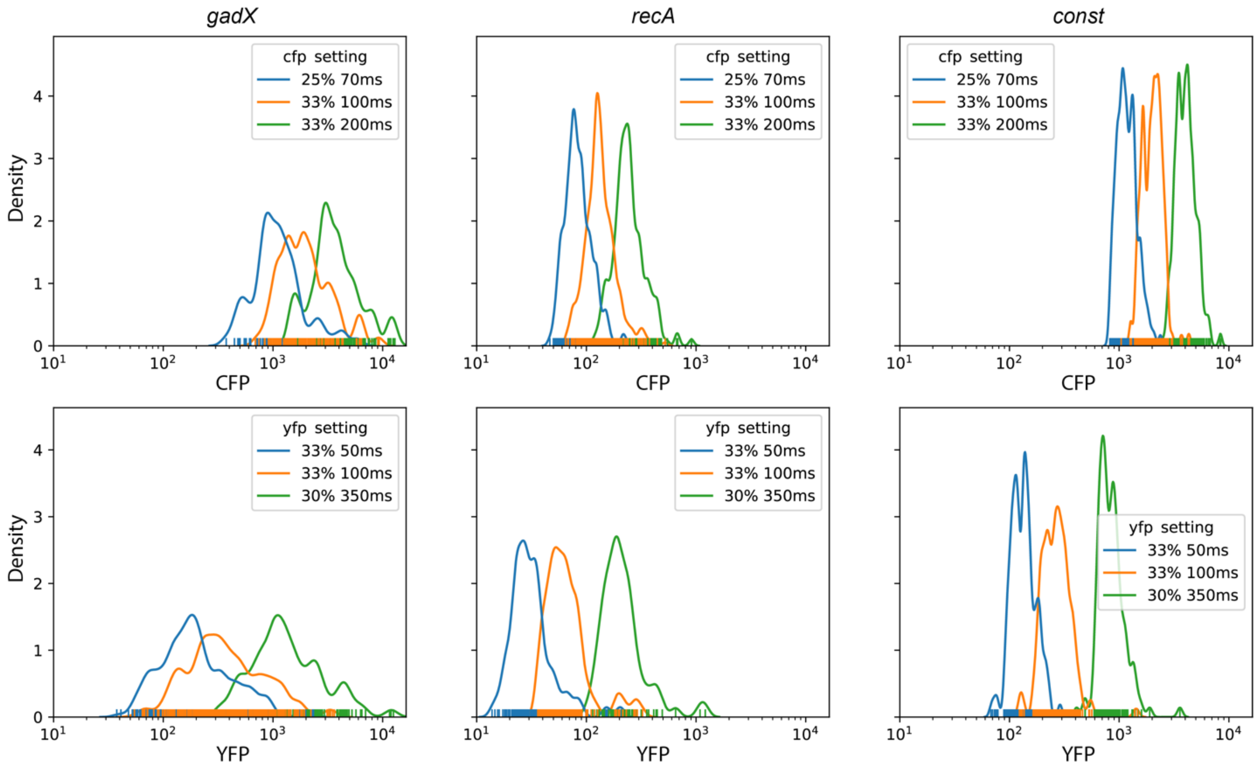

Figure S4. Effect of the imaging setting on the fluorescence population distributions. In calibration experiments, we imaged the same cell population with six different imaging settings, three for each color. We found that the fluorescence distributions translate without changing shape on a log scale, which indicates that the effect of the imaging setting is a multiplicative factor.

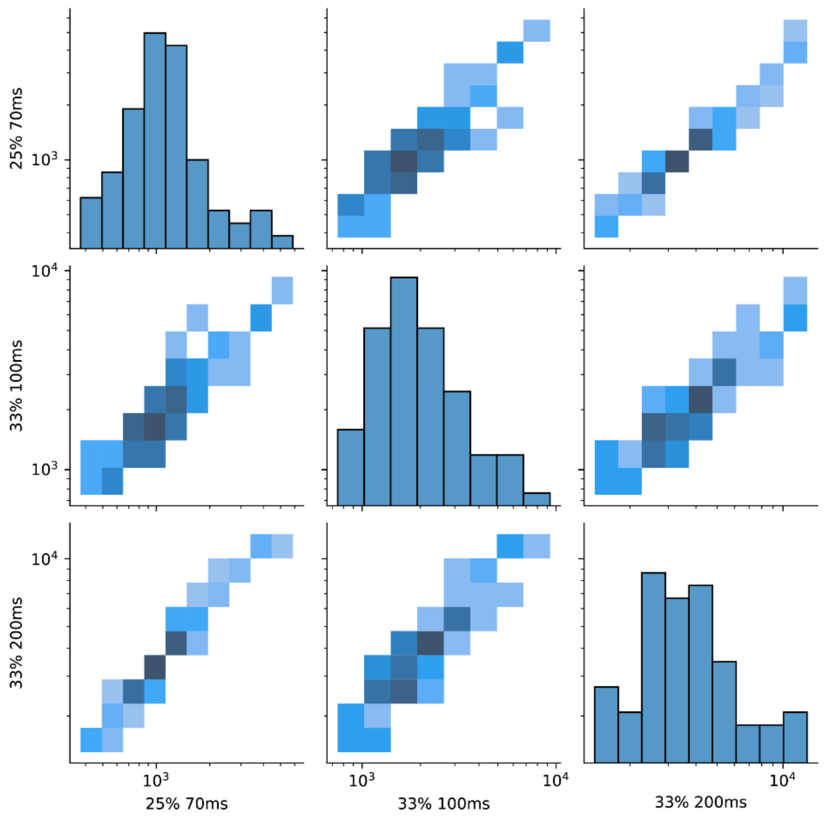

Figure S5. Effect of the imaging setting on single cell fluorescence. One-dimensional and two-dimensional histograms of the raw fluorescence values for P_{gadX}-CFP from calibration experiments. The presence of diagonal lines shows that the values across settings are largely proportional, which supports the multiplicative hypothesis even at a single-cell level. Other promoters (P_{recA}, P_{const}) and colors (YFP) are similar to this graph.

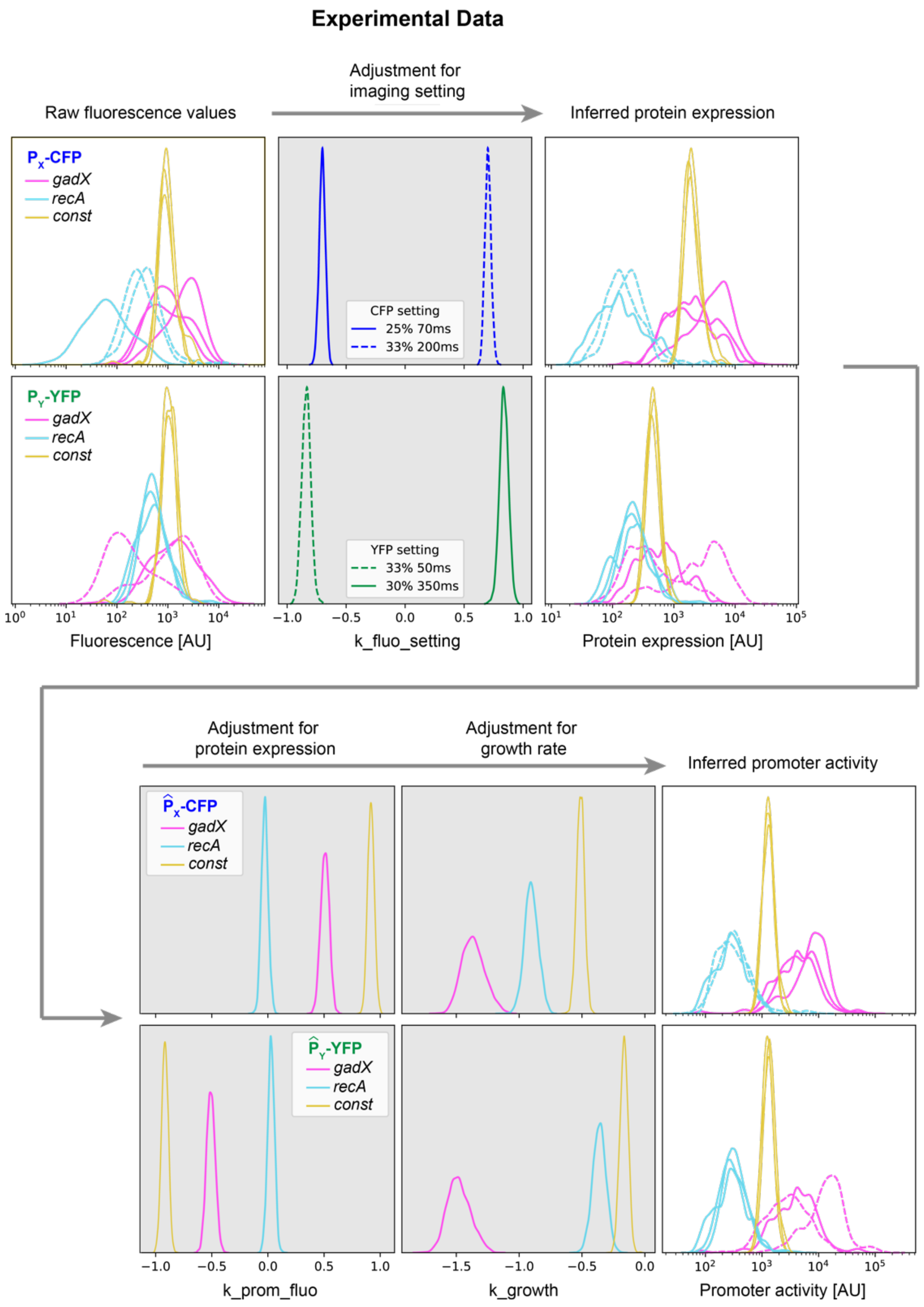

Figure S6. Transformation of raw fluorescence values to inferred promoter activity. This figure expands on Fig. 3C to show the posterior distributions of the parameters linking the raw fluorescence values, inferred protein expression, and inferred promoter activity. Refer to Methods for the details of the model.

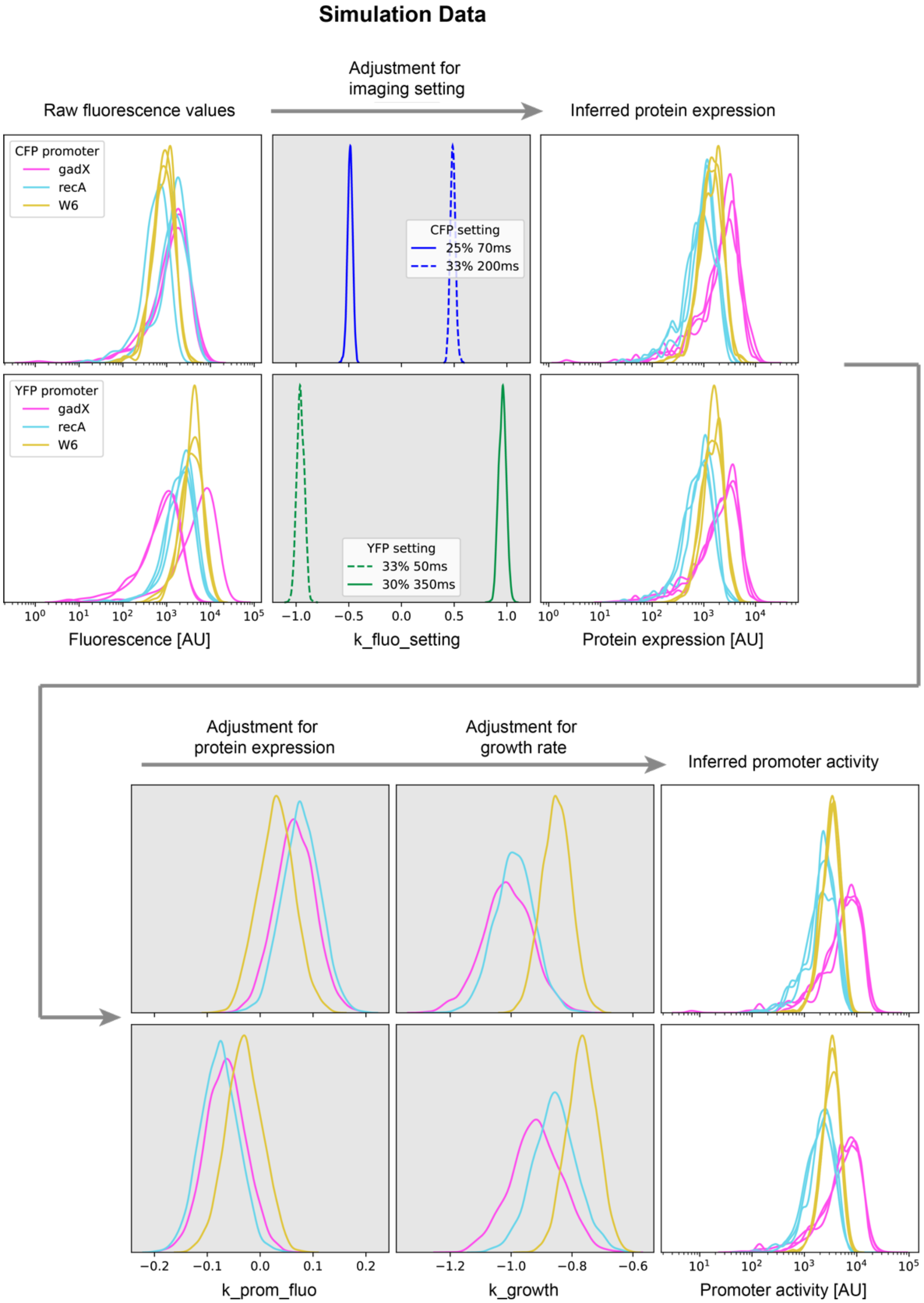

Figure S7. Transformation of raw fluorescence values to inferred promoter activity (on simulated data). Equivalent data to Fig. S6 generated with simulated data. Refer to Methods for details of the model, and to Supplementary Text for details of the simulation.

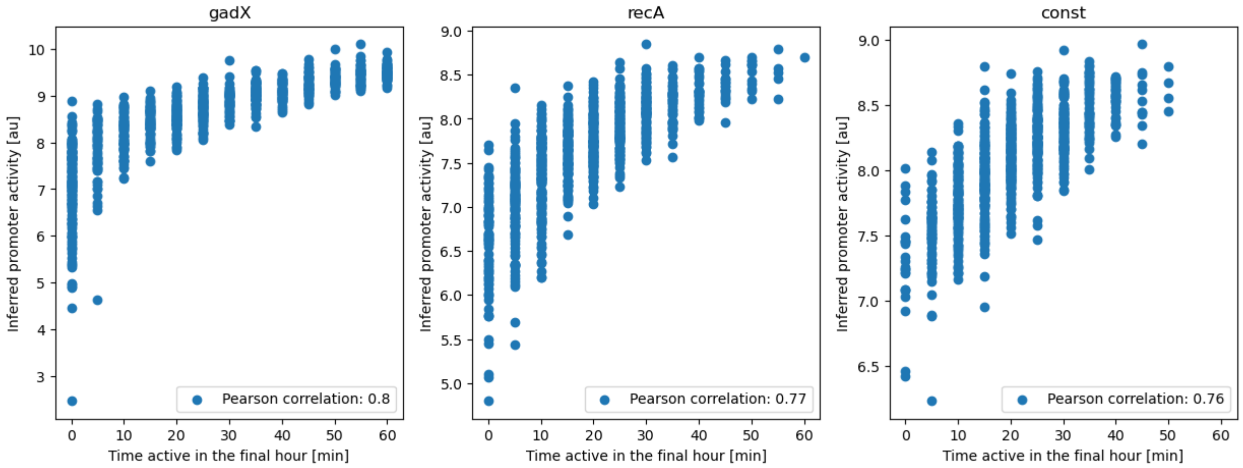

Figure S8. Test of the inferred promoter activity model on data from a stochastic simulation. This figure shows the agreement between the inferred promoter activity, and the time active in the final hour for each promoter in a simulated dataset. Refer to Supplementary Text for details of the simulation.

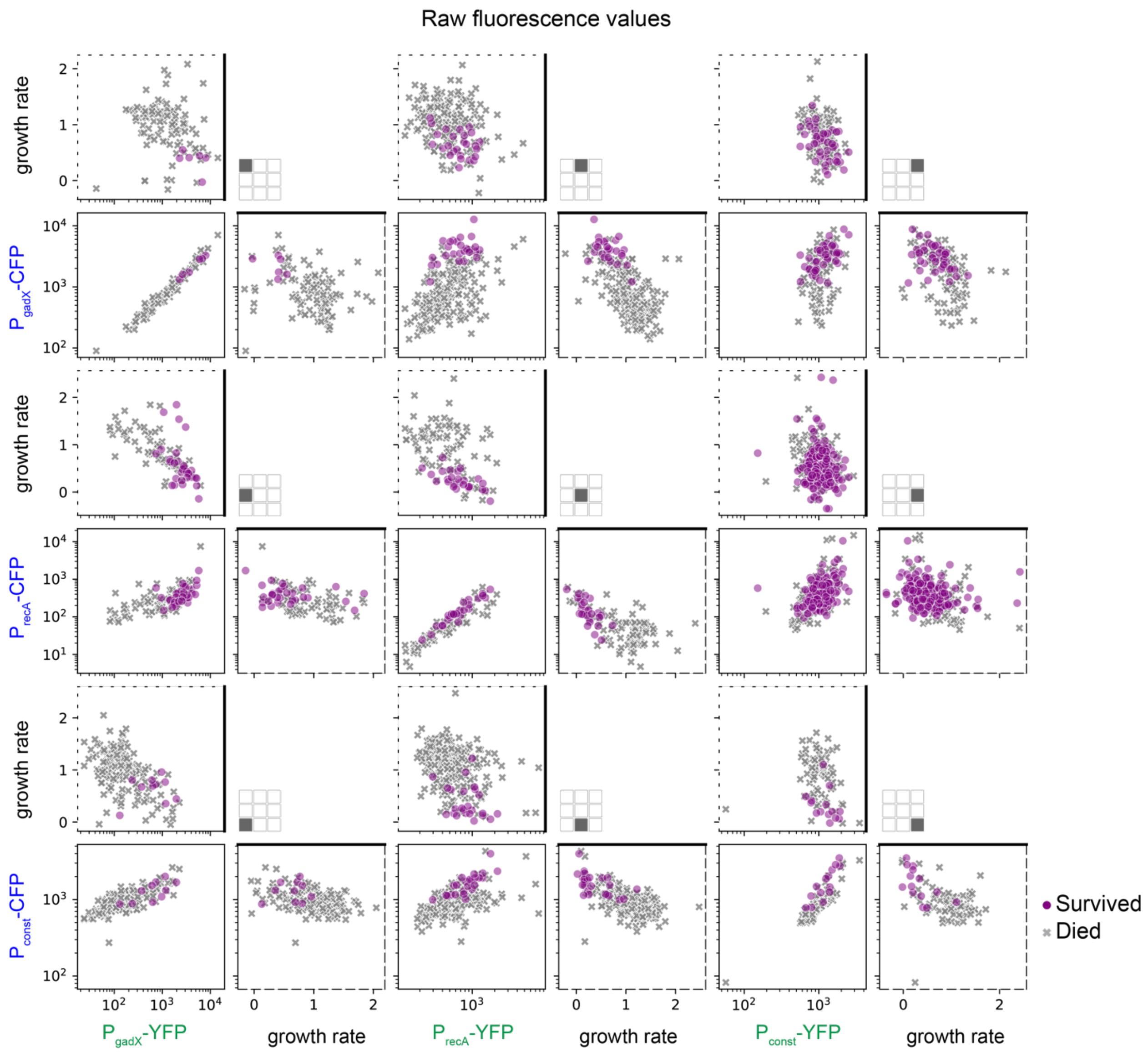

Figure S9. Relation between growth rate, raw fluorescence values, and survival. Raw data associated with Fig. 3E, before applying the corrections. Growth rate units 1/h; fluorescence units AU.

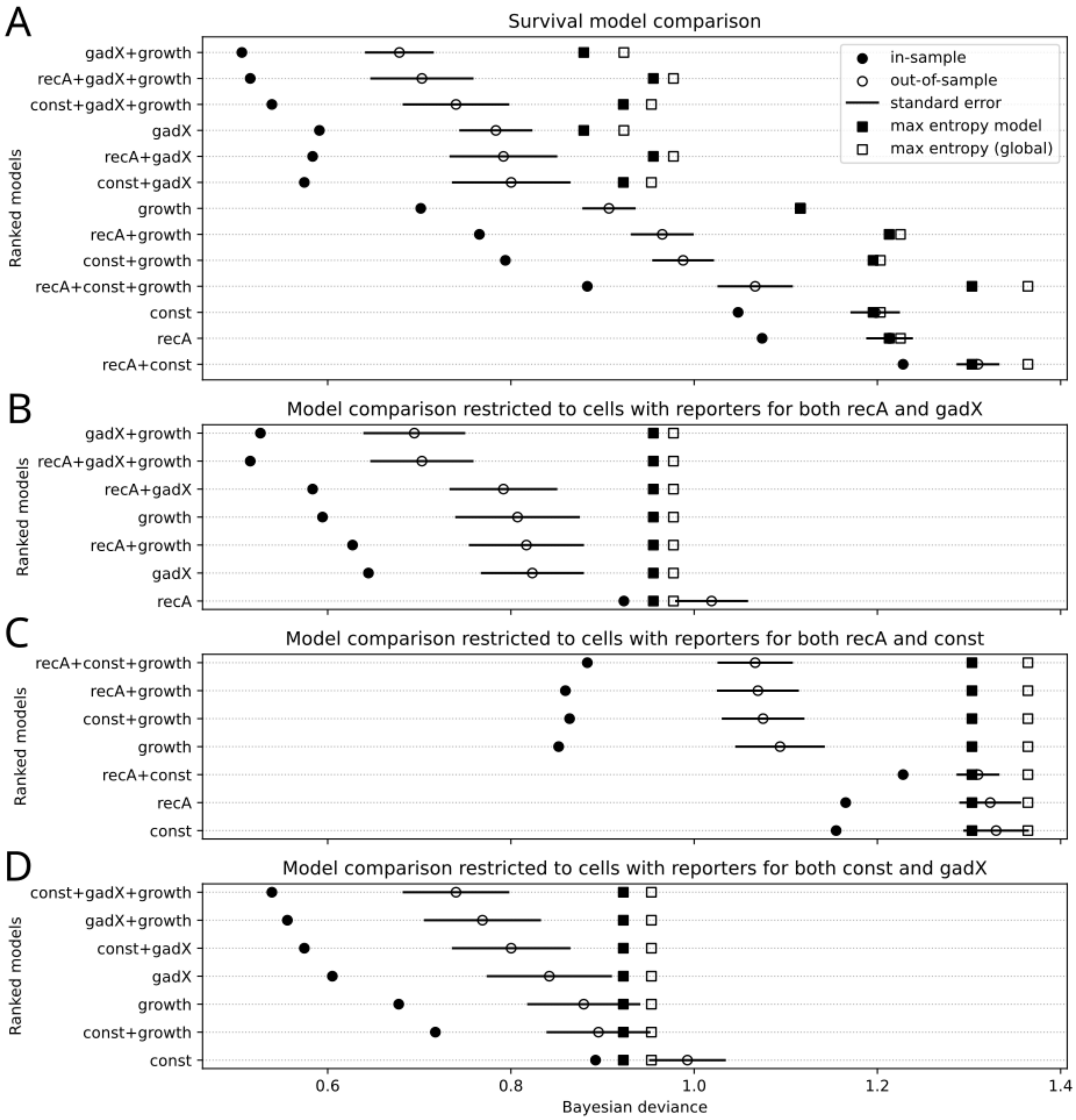

Figure S10. Assessment of the prediction accuracy of the survival models from Figure 4 in the main text. Bayesian out-of-sample deviance values derived using leave-one-out cross-validation (LOO-CV). Lower deviance values correspond to better predictions. Note that models that have $\widehat{P}_{gadX}$ and growth as predictors have lower deviance values than those that do not. **(A)** Models assessed on the largest possible dataset. For example, the $\widehat{P}_{gadX}$ model is evaluated on all cells that have at least one P_{gadX} reporter. **(B, C, D)** Models assessed on comparable datasets, as indicated in the subpanel titles. In all panels in this figure, we included a baseline maximum entropy model, which evaluates survival by flipping a coin that is biased on the distribution of surviving cells. Hollow squares: biased on the overall proportion of surviving cells, filled squares: biased on the proportion of surviving cells in this particular dataset. We found results with the Widely Applicable Information Criterion (WAIC) to be identical to LOO-CV (Methods). Further details are available in Supplementary Text.

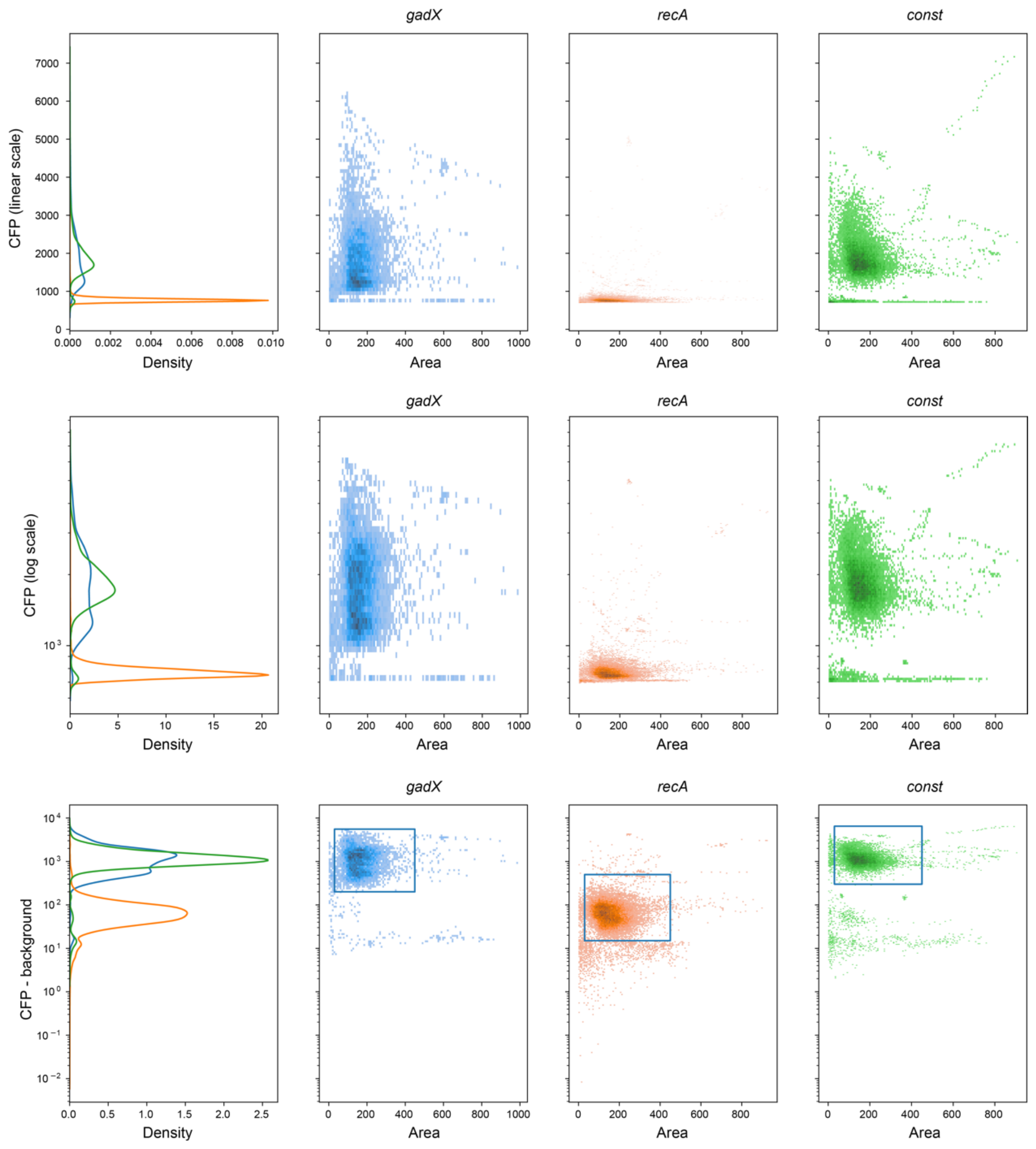

Figure S11. After background subtraction, logarithmic scale is the natural scale for fluorescence distributions. In the first row, raw fluorescence distributions as a function of cell area on a linear scale. Second row, the same data plotted with a logarithmic scale for fluorescence. Third row, the scale is logarithmic after we remove the background fluorescence. In every row, the first column is the marginal distribution of fluorescence. This figure shows the same data represented in three different ways, but illustrates that the third representation is more regular than the first two. The boxes indicate the gating values, which set the points that we retained and those that we did not. Data shown here are for P_{gadX}-CFP, P_{recA}-CFP, and P_{const}-CFP using the 25%-70ms setting, and are representative of all data.

Supplementary Tables

Table S1. Plasmids used in this study.

P_X-CFP	P_Y-YFP	Origin	Resistance Marker	Addgene Plasmid ID
P_{gadX}-CFP	P_{gadX}-YFP	pSC101	KanR	228095
P_{gadX}-CFP	P_{recA}-YFP	pSC101	KanR	228094
P_{gadX}-CFP	P_{const}-YFP	pSC101	KanR	228096
P_{recA}-CFP	P_{gadX}-YFP	pSC101	KanR	228098
P_{recA}-CFP	P_{recA}-YFP	pSC101	KanR	228097
P_{recA}-CFP	P_{const}-YFP	pSC101	KanR	228099
P_{const}-CFP	P_{gadX}-YFP	pSC101	KanR	228102
P_{const}-CFP	P_{recA}-YFP	pSC101	KanR	228101
P_{const}-CFP	P_{const}-YFP	pSC101	KanR	228100

Table S2. Microscopy imaging settings. Different settings were used to keep fluorescence within the camera detection range. Calibration experiments were conducted to correct for these different settings (Figs. S4 and S5).

P_X-CFP / P_Y-YFP	CFP imaging settings	YFP imaging settings
P_{gadX}-CFP / P_{gadX}-YFP	25%, 70ms	30%, 350ms
P_{gadX}-CFP / P_{recA}-YFP	25%, 70ms	30%, 350ms
P_{gadX}-CFP / P_{const}-YFP	25%, 70ms	30%, 350ms
P_{recA}-CFP / P_{gadX}-YFP	33%, 200ms	33%, 50ms
P_{recA}-CFP / P_{recA}-YFP	25%, 70ms	30%, 350ms
P_{recA}-CFP / P_{const}-YFP	33%, 200ms	30%, 350ms
P_{const}-CFP / P_{gadX}-YFP	25%, 70ms	33%, 50ms
P_{const}-CFP / P_{recA}-YFP	25%, 70ms	30%, 350ms
P_{const}-CFP / P_{const}-YFP	25%, 70ms	30%, 350ms

Table S3. Experimental sample sizes for each reporter strain. These same data (and sample sizes) are used for analyses across all figures in the paper. Biological replicates are independent experiments originating from separate colonies for each strain.

P_x-CFP / P_y-YFP	Number of Cells	Biological Replicates
P_{gadX}-CFP / P_{gadX}-YFP	111	3
P_{gadX}-CFP / P_{recA}-YFP	211	7
P_{gadX}-CFP / P_{const}-YFP	138	4

P_{recA}-CFP / P_{gadX}-YFP	101	3
P_{recA}-CFP / P_{recA}-YFP	117	3
P_{recA}-CFP / P_{const}-YFP	383	4
P_{const}-CFP / P_{gadX}-YFP	167	3
P_{const}-CFP / P_{recA}-YFP	294	4
P_{const}-CFP / P_{const}-YFP	104	3

Table S4. Regression coefficients: average and 94% credible intervals for the survival models in Figure 4 of the main text.

	**gadX (3%; 97%)**	**recA (3%; 97%)**	**const (3%; 97%)**	**growth (3%; 97%)**
**One variable**	0.501 (0.354; 0.664)			
		0.106 (-0.074; 0.300)		
			-0.037 (-0.287; 0.213)	
				-2.773 (-3.118; -2.450)
**Two variables**	1.161 (0.792; 1.543)	-1.578 (-2.189, -0.981)		
	1.259 (0.806; 1.686)		-1.655 (-2.241; -1.077)	
	0.588 (0.422; 0.744)			-2.454 (-3.027; -1.908)
		-0.010 (-0.313, 0.310)	-0.021 (-0.414; 0.348)	
		0.170 (-0.015; 0.375)		-2.842 (-3.215; -2.456)
			0.174 (-0.080; 0.413)	-2.692 (-3.084; -2.294)
**Three variables**	0.978 (0.607; 1.365)	-1.086 (-1.670; -0.459)		-2.221 (-3.020; -1.398)
	1.273 (0.805; 1.740)		-1.501 (-2.129; -0.900)	-1.888 (-2.733; -1.007)
		0.038 (-0.275; 0.358)	0.161 (-0.221; 0.544)	-2.683 (-3.133, -2.195)

Supplementary Movie Captions

Movie S1. Representative example of cells growing in the mother machine microfluidic device with the P_{gadX}-CFP / P_{gadX}-YFP reporter plasmid. Time is in HH:MM and scale bar is 10 µm. The mother cell in chamber 17 from the left is the cell portrayed in Fig. 2A. Ciprofloxacin addition at hour 10 is shown by the addition of media with red dye in the media flow channel. The videos continue for 18 hours after ciprofloxacin addition to display cells that survived and died after antibiotic exposure.

Movie S2. Representative example of cells growing in the mother machine microfluidic device with the P_{gadX}-CFP / P_{recA}-YFP reporter plasmid. Time is in HH:MM and scale bar is 10 µm. The mother cell in chamber 9 from the left is the cell portrayed in Fig. 2A. Ciprofloxacin addition at hour 10 is shown by the addition of media with red dye in the media flow channel. The videos continue for 18 hours after ciprofloxacin addition to display cells that survived and died after antibiotic exposure.

Movie S3. Representative example of cells growing in the mother machine microfluidic device with the P_{gadX}-CFP / P_{const}-YFP reporter plasmid. Time is in HH:MM and scale bar is 10 µm. The mother cell in chamber 13 from the left is the cell portrayed in Fig. 2A. Ciprofloxacin addition at hour 10 is shown by the addition of media with red dye in the media flow channel. The videos continue for 18 hours after ciprofloxacin addition to display cells that survived and died after antibiotic exposure.

Movie S4. Representative example of cells growing in the mother machine microfluidic device with the P_{recA}-CFP / P_{gadX}-YFP reporter plasmid. Time is in HH:MM and scale bar is 10 µm. The mother cell in chamber 9 from the left is the cell portrayed in Fig. 2A. Ciprofloxacin addition at hour 10 is shown by the addition of media with red dye in the media flow channel. The videos continue for 18 hours after ciprofloxacin addition to display cells that survived and died after antibiotic exposure.

Movie S5. Representative example of cells growing in the mother machine microfluidic device with the P_{recA}-CFP / P_{recA}-YFP reporter plasmid. Time is in HH:MM and scale bar is 10 µm. The mother cell in chamber 3 from the left is the cell portrayed in Fig. 2A. Ciprofloxacin addition at hour 10 is shown by the addition of media with red dye in the media flow channel. The videos continue for 18 hours after ciprofloxacin addition to display cells that survived and died after antibiotic exposure.

Movie S6. Representative example of cells growing in the mother machine microfluidic device with the P_{recA}-CFP / P_{const}-YFP reporter plasmid. Time is in HH:MM and scale bar is 10 µm. The mother cell in chamber 15 from the left is the cell portrayed in Fig. 2A. Ciprofloxacin addition at hour 10 is shown by the addition of media with red dye in the media flow channel. The videos continue for 18 hours after ciprofloxacin addition to display cells that survived and died after antibiotic exposure.

Movie S7. Representative example of cells growing in the mother machine microfluidic device with the P_{const}-CFP / P_{gadX}-YFP reporter plasmid. Time is in HH:MM and scale bar is 10 µm. The

mother cell in chamber 11 from the left is the cell portrayed in Fig. 2A. Ciprofloxacin addition at hour 10 is shown by the addition of media with red dye in the media flow channel. The videos continue for 18 hours after ciprofloxacin addition to display cells that survived and died after antibiotic exposure.

Movie S8. Representative example of cells growing in the mother machine microfluidic device with the P_{const}-CFP / P_{recA}-YFP reporter plasmid. Time is in HH:MM and scale bar is 10 µm. The mother cell in chamber 11 from the left is the cell portrayed in Fig. 2A. Ciprofloxacin addition at hour 10 is shown by the addition of media with red dye in the media flow channel. The videos continue for 18 hours after ciprofloxacin addition to display cells that survived and died after antibiotic exposure.

Movie S9. Representative example of cells growing in the mother machine microfluidic device with the P_{const}-CFP / P_{const}-YFP reporter plasmid. Time is in HH:MM and scale bar is 10 µm. The mother cell in chamber 10 from the left is the cell portrayed in Fig. 2A. Ciprofloxacin addition at hour 10 is shown by the addition of media with red dye in the media flow channel. The videos continue for 18 hours after ciprofloxacin addition to display cells that survived and died after antibiotic exposure.

Supplementary Text

Assessing the accuracy of the promoter activity inference model

In order to test whether the Bayesian inference model used in Fig. 3C to normalize the data and extract the promoter activity is accurate, we replicated all our data in silico. To simulate promoter activity and reporter expression, we used the classical telegraph model[1–3] that we simulated with the rebop library[4], a Rust implementation of the Gillespie algorithm[5]. We focused on three possible promoters per cell, which we called *gadX*, *recA*, and *const*, to match those we measured experimentally. We also employed two different reporters, which we refer to as CFP and YFP to match our experiments. For each of the nine possible combinations of dual reporters, we simulated 175 cells, leading to a total of 1575 cells, comparable to the 1626 cells in the experimental dataset (Table S3). Note that we did not need to consider cell death or antibiotic exposure to test the promoter activity inference. To make growth rate fluctuate stochastically, we model growth rate as if it were a molecular species subject to immigration-death reactions. This growth rate determines how fast the cell volume grows at each time step, and upon reaching a given volume, cells divide into two cells of equal volume (half the splitting volume), dividing their contents with a Binomial distribution. The model that we used was the following:

**Cells with reporters for two distinct promoters**		**Cells with reporters for the same promoter**	
**Reaction**	**Rate constant**	**Reaction**	**Rate constant**
$\emptyset \longrightarrow GR$	$\lambda_{GR} \cdot GR_0$	$\emptyset \longrightarrow GR$	$\lambda_{GR} \cdot GR_0$
$GR \longrightarrow \emptyset$	λ_{GR}	$GR \longrightarrow \emptyset$	λ_{GR}
$Poff_1 \longrightarrow Pon_1$	kon_1	$Poff \longrightarrow Pon$	kon
$Poff_2 \longrightarrow Pon_2$	kon_2		
$Pon_1 \longrightarrow Poff_1$	$koff_1$	$Pon \longrightarrow Poff$	$koff$
$Pon_2 \longrightarrow Poff_2$	$koff_2$		
$Pon_1 \longrightarrow Pon_1 + mRNA_1$	km_1	$Pon \longrightarrow Pon + mRNA_1$	km_1
$Pon_2 \longrightarrow Pon_2 + mRNA_2$	km_2	$Pon \longrightarrow Pon + mRNA_2$	km_2
$mRNA_1 \longrightarrow mRNA_1 + Fluo_1$	kp_1	$mRNA_1 \longrightarrow mRNA_1 + Fluo_1$	kp_1
$mRNA_2 \longrightarrow mRNA_2 + Fluo_2$	kp_2	$mRNA_2 \longrightarrow mRNA_2 + Fluo_2$	kp_2
$mRNA_1 \longrightarrow \emptyset$	λ_{mRNA_1}	$mRNA_1 \longrightarrow \emptyset$	λ_{mRNA_1}
$mRNA_2 \longrightarrow \emptyset$	λ_{mRNA_2}	$mRNA_2 \longrightarrow \emptyset$	λ_{mRNA_2}
$Fluo_1 \longrightarrow \emptyset$	λ_{Fluo_1}	$Fluo_1 \longrightarrow \emptyset$	λ_{Fluo_1}
$Fluo_2 \longrightarrow \emptyset$	λ_{Fluo_2}	$Fluo_2 \longrightarrow \emptyset$	λ_{Fluo_2}

GR is a fictive chemical species that helps simulate growth rate fluctuations (see paragraph below). Pon and Poff represent a promoter in the active and inactive state, respectively. The sum of Pon and Poff is always one, for a given promoter (i.e. the same promoter cannot be both active and inactive at the same time in the same cell). mRNA represent mRNAs and Fluo represent fluorescent proteins. The indices 1 and 2 refer to which promoter or reporter are present in a given cell (see tables below).

These reactions simulate random growth rate fluctuations with the variable GR taking integer values of average GR_0. To convert these values into a proper growth rate we divide them by GR_0

and the resulting value is the instantaneous growth rate in hr^{-1}. For example, with our parameters, a cell with GR = 8 is elongating at a rate 1 hr^{-1}.

The values of the parameters that we used are listed below. These values were selected to approximately match the simulation with our experimental data and inference results, but should not be construed as exact values for these parameters.

**Common parameters**	
λ_{GR}	0.1 hr^{-1}
GR_0	8

**Promoter-related parameters**	***gadX***	***recA***	***const***
kon	1.5 hr^{-1}	3 hr^{-1}	5 hr^{-1}
$koff$	2 hr^{-1}	7 hr^{-1}	10 hr^{-1}
km	200 hr^{-1}	100 hr^{-1}	150 hr^{-1}

**Reporter-related parameters**	**CFP**	**YFP**
kp	700 hr^{-1}	500 hr^{-1}
λ_{mRNA}	15 hr^{-1}	10 hr^{-1}
λ_{Fluo}	0.25 hr^{-1}	0.5 hr^{-1}

Additionally, to simulate different imaging settings, we multiplied the simulated fluorescence values by a gain representing the exposure time. We used the same exposures as in the experiments (Table S2).

**Imaging-related parameters**	**CFP**		**YFP**	
	**25% 70ms**	**33% 200ms**	**33% 50ms**	**30% 350ms**
gain	0.7	2.0	0.5	3.5

In the simulation, every cell is initialized with a fixed initial condition (Pon = 1, Poff = 0, mRNA = 0, Fluo = 0, GR = GR_0) and is given a 35-hour burn-in time (during which it grows and divides) to reach a random and biologically plausible state. Then, we collect the two single-cell fluorescence values (multiplied by the appropriate gains), and their growth rate averaged over the previous hour (as in the experiments). We then analyzed these data as if they were the experimental data. First, we obtained Fig. S7 (to be compared to Fig. S6 obtained from the experimental data). We first noticed that the values determined for the optical setting adjustments matched the gains that we provided (CFP: log(2.0 / 0.7) / 2 = 0.52, YFP: log(3.5 / 0.5) / 2 = 0.97). Next, focusing on the inferred promoter activity distributions (last panel of Fig. S7), we observed that we could modify the average of the distributions by modifying the ratios kon/koff, while we could modify their widths by changing kon and koff by the same factor. Note that these observations correspond to the relative ordering and width of the *gadX*, *recA*, and *const* distributions, relatively to their kon and koff parameters.

Next, we asked whether the inferred promoter activity was accurately representing single-cell promoter activity. To estimate this, we compared the model-inferred promoter activity to the duration that the promoter was activated during the last hour of the simulation. To estimate this duration, we sampled the promoter activity every 5 minutes and counted the number of times that we saw it active during the last hour of the simulation. Plotting the inferred promoter activity as a function of the actual time active, we found good agreement between the inferred and actual values, with Pearson correlation coefficients between 0.76 and 0.8 depending on the promoter (Fig. S8). These results confirm that our approach for calculating promoter activity is reasonable.

Assessing the predictive accuracy of the survival models

To assess the predictive accuracy of the different cell survival models shown in Figure 4 of the main text, we computed their Bayesian deviance, a notion related to the loss in machine learning. Briefly, if a model predicts a probability of survival p for a cell that survives, the contribution of this observation to this model's total deviance is $-2\log(p)$, while if the cell actually dies, the deviance is $-2\log(1-p)$. (Note that our approach differs slightly from standard practice because in this analysis we consider the average deviance per observation instead of the sum of the observation's deviances. This modification allows us to compare models on different datasets, with different numbers of observations.) A model which correctly predicts all survivals and deaths with certain probability would have a deviance of 0; positive deviance values occur when there is uncertainty or prediction error, as is commonly the case. As a baseline for deviance, we used a maximum entropy model, which predicts cell survival by flipping a biased coin, where the bias corresponds to the overall proportion of surviving cells in the full dataset. Although this is a poor model, it corresponds to the best performance that is possible without looking at the individual cells. Because of this, it establishes a realistic upper limit, which better models are expected to outperform, i.e. have lower deviance than.

Evaluating the Bayesian deviance on the observations gives an assessment of the predictive accuracy of the model on in-sample data, i.e. data that the model has been trained on. This evaluation shows that $\hat{P}_{gadX}$ and growth are important predictors of cell survival, resulting in the lowest deviance values (Fig. S10, filled circles).

However, a more rigorous test for the model predictive power is on out-of-sample data, i.e. data that it was not trained on. A common approach for this is leave-one-out cross-validation (LOO-CV), where for each observation, a dataset is created without this observation, the model is trained on this dataset, and the performance of the model on this observation is evaluated. While this requires an extensive computational effort, an approximation exists to compute this without the need to retrain the model: Pareto-smoothed importance sampling (PSIS). With this method, we obtained N deviances, one for each observation, as if this observation were new to the model. We plotted the average of these deviances and the standard error on the mean (Fig. S10, hollow circles). We found that this out-of-sample estimation was worse than the in-sample one (as expected), but that this did not change the conclusions that $\hat{P}_{gadX}$ and growth were the best predictors of cell survival.

Finally, because each model is evaluated on a different dataset with varying proportions of surviving cells, this makes it difficult to compare the models directly. To avoid this issue, we next

limited model comparisons involving two given reporters to the cells that just have these two. For example, in Fig. S10B the cells considered are only the ones that have P_{gadX} and P_{recA} in any order, while in Fig. S10A, the *gadX* model was evaluated on all the cells with a P_{gadX} reporter, regardless of the other reporter. Overall, these data confirm that $\hat{P}_{gadX}$ and growth rate are good predictors of survival, while $\hat{P}_{recA}$ and $\hat{P}_{const}$ are poor predictors.

Supplementary References

1. Ko, M. S. H. A stochastic model for gene induction. *J. Theor. Biol.* **153**, 181–194 (1991).
2. Peccoud, J. & Ycart, B. Markovian Modeling of Gene-Product Synthesis. *Theor. Popul. Biol.* **48**, 222–234 (1995).
3. Paulsson, J. Models of stochastic gene expression. *Phys. Life Rev.* **2**, 157–175 (2005).
4. Andreani, V. (2025). rebop (Version 0.9.1) [Computer software]. https://github.com/Armavica/rebop
5. Gillespie, D. T. Exact stochastic simulation of coupled chemical reactions. *J. Phys. Chem.* **81**, 2340–2361 (1977).
